# Pancreatic MicroRNAs in *Ictidomys tridecemlineatus* Associated with Metabolic Diseases: Nature’s Insights into Important Biomarkers

**DOI:** 10.3390/biom15050616

**Published:** 2025-04-23

**Authors:** Olawale O. Taiwo, Saif Rehman, Kenneth B. Storey

**Affiliations:** Department of Biology, Carleton University, Ottawa, ON K1S 5B6, Canada; olawaletaiwo@cmail.carleton.ca (O.O.T.); saifrehman@cmail.carleton.ca (S.R.)

**Keywords:** microRNA, hibernation, pancreatic tissue, metabolic rate suppression, glucose homeostasis, metabolic disease

## Abstract

Hibernation involves a profound metabolic rate depression (MRD) that enables certain species to survive prolonged periods of low energy availability. The thirteen-lined ground squirrel uses MRD to arrange cellular and biochemical pathways which suppress nonvital genetic and cellular pathways to conserve internal energy while preserving all essential processes. This study investigates the role of microRNAs (miRNAs) in controlling key signaling pathways and cellular processes in pancreatic tissue during hibernation. Using next-generation sequencing and broad genomic analysis, we analyzed and identified seven differentially expressed miRNAs (miR-29a-3p, miR-22-3p, miR-125-5p, miR-200a-3p, miR-328-3p, miR-21-5p, and miR-148-3p) in the pancreas of hibernating 13-lined ground squirrels (*Ictidomys tridecemlineatus*). Our findings reveal that these miRNAs regulate pathways involved in glucose homeostasis, including insulin secretion and metabolic regulation, contributing to the unique adaptations of hibernation. These insights advance our understanding of the molecular adaptations underlying hibernation and may have implications for therapeutic strategies targeting metabolic disorders such as diabetes.

## 1. Introduction

Following periods of prolonged cold temperatures and food shortages, some mammals may undergo a state of torpor, a physiological state which is characterized by periods of reduced metabolic activity. During hibernation, an animal’s body temperature drops significantly, and its metabolic processes slow down to conserve energy [1]. The 13-lined ground squirrel (*Ictidomys tridecemlineatus*), a species native to North America, is one of the best-studied hibernators. This mammal undergoes seasonal hibernation during which it cycles between periods of torpor, during which metabolic rates are remarkably reduced, and brief arousal periods, in which metabolism increases [2]. Prior to hibernation, 13-lined ground squirrels undergo several preparatory changes. They accumulate fat reserves through hyperphagia to sustain them during the winter [3]. The squirrels then reduce activity and build a burrow for hibernation. Physiologically, their body temperature drops to near ambient levels, entering *torpor*, with a dramatic decrease in metabolic rate, heart rate, and respiration. During torpor, their body temperature can drop to as low as 2–5 °C, and their heart rate slows to as few as 4–6 beats per minute [1]. Hormonal changes, including shifts in insulin and thyroid hormone levels, help regulate their metabolism. The squirrels periodically arouse from torpor in a process called interbout arousal, which may help maintain immune function and tissue repair. These adaptations, along with the modulation of their immune system and metabolic pathways, ensure their survival and readiness for reproduction once they emerge from hibernation in spring [2,3,4]. While majorly repressing all energetic cellular processes, metabolic rate depression (MRD) upregulates metabolic pathways that will maintain tissue homeostasis. For example, it has been shown that hibernators epigenetically control the transcriptional regulation of DNA access regulatory enzymes like kinases, regulatory proteins (antioxidants, chaperones, and cryoprotective) [5,6,7]. Understanding how these metabolic shifts are coordinated at the molecular level could offer insights into advancing health technologies that could elongate organ preservation for transplant purposes and improve treatments for metabolic diseases like diabetes.

One class of regulatory molecules thought to play a crucial role in hibernation are microRNAs (miRNAs) [8]. MiRNAs are small, non-coding RNA molecules that regulate gene expression by binding to complementary sequences on target mRNAs [9,10], typically leading to their degradation or suppression of translation. MiRNAs are involved in virtually every biological process, including development, cell differentiation, and, notably, metabolism [11]. In hibernating species, miRNAs are believed to modulate the complex molecular networks that regulate energy homeostasis, glucose metabolism, and cellular stress responses during periods of metabolic suppression. While previous studies have looked at the role of miRNAs across different organs during hibernation and similar stressors [12,13,14], their specific roles in regulating the pancreas and its functions during hibernation remain unknown.

The pancreas is a critical organ in metabolic regulation, particularly in glucose homeostasis. It controls blood glucose levels through the secretion of insulin and glucagon, hormones that balance energy supply and demand [15]. During hibernation, animals experience significant fluctuations in glucose levels as they cycle between periods of metabolic suppression and reactivation [16]. While the broader molecular adaptations of tissues involved in glucose metabolism, such as the liver and muscle, have been studied in hibernators [16], little attention has been given to how the pancreas might adapt to these extreme changes. Specifically, it remains unclear how miRNA expression in the pancreas changes during hibernation and how these changes might influence pancreatic function, particularly insulin secretion and glucose regulation.

Recent studies have highlighted the potential of miRNAs as regulators of glucose metabolism in various species, including humans [17,18]. In mammals, miRNAs have been shown to regulate key genes involved in insulin production and secretion, as well as in response to insulin [19,20]. However, their role in hibernating species, particularly in organs like the pancreas that are central to metabolic control, remains underexplored. Given the dynamic nature of pancreatic function during hibernation, miRNAs may be key players in adapting glucose regulation to the extreme fluctuations seen in this metabolic state. Therefore, the primary objective of this study is to identify and characterize the miRNA expression profiles in the pancreas of hibernating 13-lined ground squirrels, comparing active and torpid states. We hypothesize that the expression of certain miRNAs in the pancreas will be significantly altered during hibernation, reflecting changes in metabolic regulation that are necessary for maintaining energy balance and glucose homeostasis. By understanding how miRNAs contribute to these adaptations, this research could provide valuable insights into the molecular mechanisms underlying hibernation and contribute to broader metabolic research, including the regulation of insulin and glucose in both health and disease. A comprehensive bioinformatic analysis was used to identify different miRNAs alongside the molecular processes that are altered after mature miRNAs were screened out following a next-generation sequencing (NGS) carried out on the RNA-seq dataset.

## 2. Materials and Methods

### 2.1. Animal Treatments and Tissues

Ground squirrel males (*Ictidomys tridecemlineatus*) within a weight of 150–300 g were caught in the wild by a United States Department of Agriculture licensed trapper (TLS Research, Bloomingdale, IL, USA) and transferred to the laboratory of Dr. J.M. Hallenback at the Animal Hibernation Facility, National Institute of Neurological Disorders and Stroke (NINDS) (NIH, Bethesda, MD, USA) where the animal experiments were conducted. Animal housing and experimental protocol were approved by the NINDS animal care and use committee (ACUC). Individual 13-lined ground squirrels were tagged with a sensor chip (IPTT-300; Bio Medic Data Systems, Seaford, DE, USA) administered subcutaneously under 5% isoflurane anesthesia and kept individually in a cage at 21 °C. The animals received standard rodent meals and fluid ad libitum until they had enough lipid stores to go into hibernation. Animals that weighed between 220 and 240 g were placed in the hibernacula and were randomly allocated to control or hibernation groups. To enable a natural transition into torpor, animals were transferred to an environmental chamber at 5 °C in constant darkness. Body temperature (Tb), time, and respiration rate were observed to determine sampling points, as previously described [21]. The control ground squirrels were housed in a cold room and had a Tb of 37 °C at the time of euthanasia. The stress condition (late torpor) contained animals that were continuously in deep torpor for at least 5 days with Tb values of 5–8 °C. All 13-lined ground squirrels had been through torpor-arousal bouts prior to euthanasia by decapitation and tissue sampling, as previously described [13,22]. Tissue samples were shipped to Carleton University on dry ice and then stored at −80 °C until use.

### 2.2. RNA Isolation and Small RNA Sequencing

This was carried out following a method previously described in detail by [13]. Briefly, pancreatic tissues (70 mg) from four control (euthermic) and four late torpid 13-lined ground squirrels were homogenized in 1 mL of TRIzol reagent (Invitrogen, Cat. No. 15596–018, Waltham, MA, USA) using a Polytron homogenizer. After adding 200 μL of chloroform, the samples were mixed and incubated at room temperature for 5 min, followed by centrifugation at 10,000 rpm for 15 min at 4 °C. The aqueous phase was carefully transferred, and the extraction was repeated to minimize lipid contamination. RNA was precipitated by adding 500 μL of isopropanol and incubating at room temperature for 10 min. Following centrifugation (10,000 rpm for 10 min at 4 °C), the RNA pellet was washed with 70% ethanol, centrifuged again, air-dried, and resuspended in 50 μL of RNase-free water. RNA quality was assessed using a BioTek Take3 multi-volume plate and spectrophotometer (260/280 nm ratio). Integrity was confirmed via 1% agarose gel electrophoresis with SYBR-Green staining, ensuring clear 28S and 18S rRNA bands.

Small RNA sequencing was performed at the Michael Smith Genome Sciences Centre (Vancouver, BC, Canada) using the Illumina NextSeq500 platform (San Diego, CA, USA), generating single-end 75 base reads. Library quality was assessed with Qubit and Agilent DNA 1000 Series II assays prior to sequencing.

### 2.3. Data Processing

Using this accession number (PRJNA1219602), raw sequences received from the RNA-seq data can be obtained from the SRA database. Raw reads were analyzed using R as previously described by [23]. Firstly, the sequence quality was determined using a FastQ Quality Filter analysis. Following that, Cutadapt version 0.10.1 was used to remove adapters, the 6 random nucleotides between the adapters and the small RNA sequences, and any reads shorter than 10 nucleotides in length, using a quality cutoff of 20. We used Bowtie’s default settings to align trimmed sequences with a library of small non-coding RNA sequences obtained from Rfam and piRNABank databases [24,25].

Sequence alignment coinciding with negative reference library were filtered out, and others were aligned with a library of mature miRNA sequences from all organisms, obtained from miRBase databank version 22 [26]. Only miRNA sequences that matched perfectly to the reference library, with a seed sequence length of 20 nucleotides, were included in the analysis. The SAMtools program, run within a UNIX shell, was used to index each read count alongside its miRNA identity in the SAM format, allowing for further analysis using the R platform [27]. miRNAs with fewer than 10 reads were excluded from the analysis. Data from four biological replicates each of control and torpid animals (*n* = 4 per group) were used in the comparison.

Data normalization and figure generation were carried out using the RBioArray package in R. This package employs the limma method to normalize RNA-seq read counts using log2-transformed counts per million (logCPM). The voom method was then applied to calculate the variance of each normalized observation [28].

### 2.4. Differential Expression Analysis

The RBioArray R package (version #0.5.6) was used to analyze the normalized and filtered miRNA expression data, incorporating functions from other R packages, including Limma [29]. To explore the patterns of differential expression, the Ward method was used to cluster miRNAs based on their expression profiles across all samples [30].

FDR-adjusted values were used in-place of the regular *p*-values for *p*-values less than 0.05. Only the differentially expressed dataset used the regular *p*-values. The GO terms were obtained from the GO.db library and mapped to the GO datasets based on their respective GO terms. Matching GO terms resulted in 14.35%, 18.28%, and 30.08% respective mismatches in the BP, CC, and MF terms. REVIGO clustering algorithm was used to summarize the GO terms in each GO dataset through the API.

To clean the dataset, the data filtering codeblocks were written to filter out tissues that are unrelated to the study, to remove terms relating to cancer since everything miRNA changes in cancer and the terms are represented by proxy. Terms beginning with “positive regulation of...” and “negative regulation of...” were also filtered to reduce the cluster. Applying these filters resulted in 855, 120, and 113 rows, respectively, for the Biological Process, Cellular Compartment, and Molecular Function term labels. Upon creating the cluster, subsets were created and grouped where Representative Terms matched or the TermID matched the representative value. Clusters were cleaned based on the terms as they conform with literature. Post-clustering and editing, the Supplementary Files were generated for all terms. The terms in each dataset are grouped into three—unchanged (*p*-value (or FDR adjusted) > 0.05), downregulated (*p*-value (or FDR adjusted) < 0.05 and a model coefficient [logfc] < 0), and upregulated (*p*-value (or FDR adjusted) < 0.05 and logfc > 0). Each of these datasets were represented in Volcano Plots using the ggplot2 R library.

## 3. Results

### 3.1. Analysis of Differentially Expressed miRNAs

**Figure 1:**
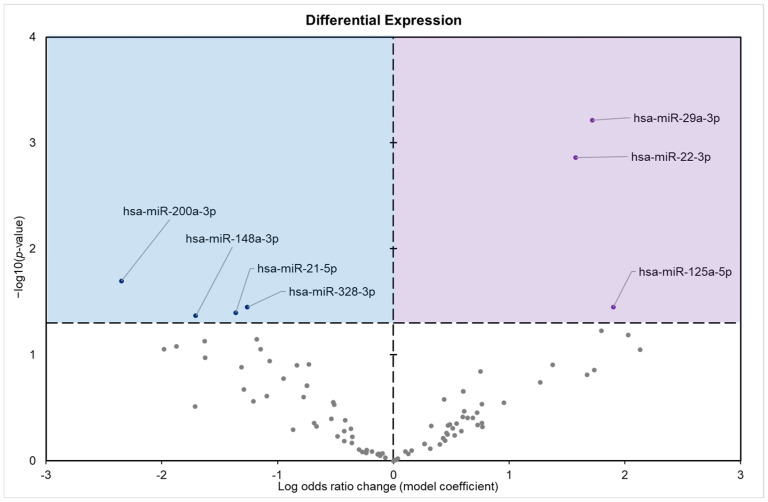
Differential expression analysis of conserved miRNAs in the pancreatic tissue of *I. tridecemlineatus*. Volcano plot of the differential expression of conserved miRNAs showing −log_10_(*p* value) versus the log_2_(fold change) for individual miRNAs in pancreas from hibernating squirrels versus the control group. Enriched terms are considered significantly altered if the FDR adjusted *p*-value is <0.05 and fold change > 1.5. Negative model coefficients depicting decreased expression are shown as blue circles in the blue-shaded box (**left**), whereas coefficients depicting increased expression are shown by purple dots in the purple-shaded box (**right**), and grey points represent non-significant miRNAs.

A total of 94 mature miRNAs were differentially expressed in the pancreas of torpid 13 lined ground squirrel. A total of seven were seen to be statistically significant (*p*-value < 0.05) during hibernation compared with euthermic control (Figure 1). Precisely, four miRNAs (miR-200a-3p, miR-328-3p, miR-21-5p, and miR-148a-3P) were downregulated, and three miRNAs (miR-29a-3p, miR-22-3p, and miR-125a-5p) were upregulated (Figure 1).

### 3.2. Gene Set Analysis

**Figure 2:**
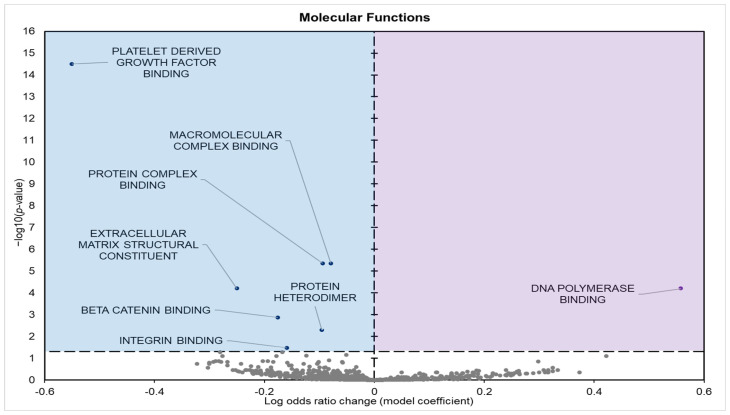
Volcano plot demonstrating the enrichment of GO molecular function (GO MF) through a logistic regression-based gene set analysis of the mRNAs predicted to interact with differentially expressed (DE) miRNAs in squirrel pancreas during hibernation. Enriched terms are considered significantly altered if the FDR-adjusted *p*-value is <0.01 and fold change > 1.5. All other information is presented as in Figure 1.

Gene enrichment analyses of GO terms for molecular functions (GO MF) identified eight significantly affected terms in the torpid pancreas of 13LGS: seven negative model and one positive model coefficients (Figure 2).

**Figure 3:**
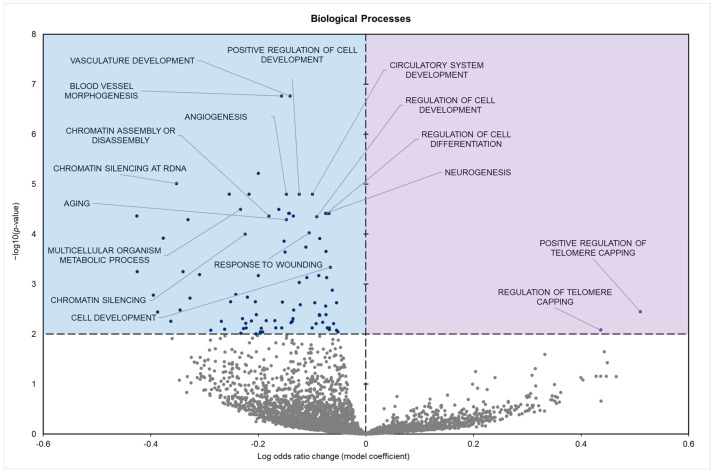
Volcano plot demonstrating the enrichment of GO biological processes (GO BPs) through a logistic regression-based gene set analysis of the mRNAs predicted to interact with DE miRNAs in squirrel pancreas during hibernation. Enriched terms are considered significantly altered if the FDR-adjusted *p*-value is <0.01 and fold change > 1.5. All other information is presented as in Figure 1.

Biological processes (GO BPs) showed 92 significantly enriched terms when comparing euthermic controls to hibernating groups; 90 had negative model coefficients, whereas 2 had positive coefficients (Figure 3).

**Figure 4:**
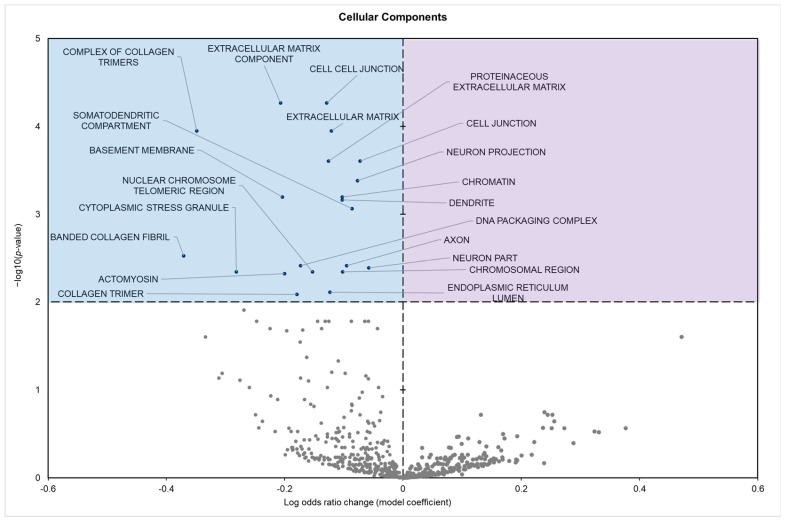
Volcano plot demonstrating the enrichment of GO cellular components (GO CCs) through a logistic regression-based gene set analysis of the mRNAs predicted to interact with DE miRNAs in squirrel pancreas during hibernation. Enriched terms are considered significantly altered if the FDR-adjusted *p*-value is <0.01 and fold change > 1.5. All other information is presented as in Figure 1.

The cellular components showed 21 significantly enriched terms; all of which were positive model coefficients (Figure 4).

**Figure 5:**
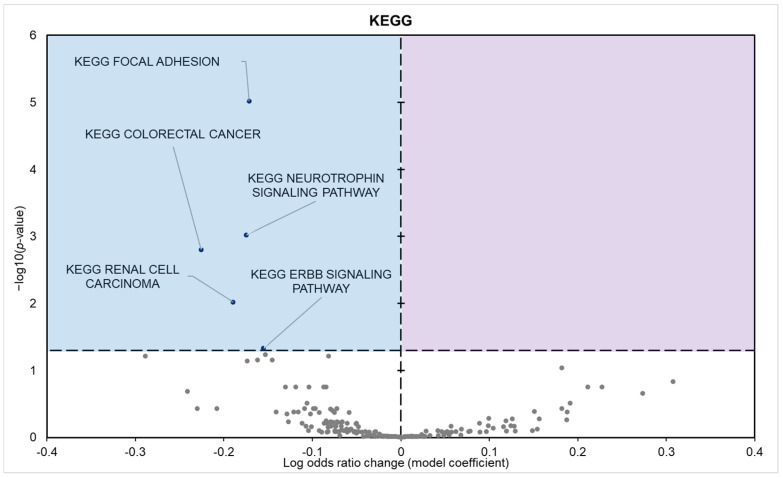
Volcano plot demonstrating the enrichment of KEGG pathways through a logistic regression-based gene set analysis of the mRNAs predicted to interact with DE miRNAs in 13-lined ground squirrel pancreas tissue during hibernation. The KEGG pathway is considered significantly altered if the FDR-adjusted *p*-value < 0.05. All other information is presented as in Figure 1.

KEGG enrichment analysis found five statistically enriched pathways in samples from hibernating squirrels, all of which depicted negative model coefficients (Figure 5). GO and KEGG term enrichment was predominantly focused on processes related to metabolic regulation, cell signaling, and cancer-related pathways.

## 4. Discussion

This study revealed a significant upregulation of three miRNAs (miR-29a-3p, miR-22-3p, and miR-125a-5p) and concurrent downregulation of four miRNAs (miR-200a-3p, miR-148a-3p, miR-21-5p, and miR-328-3p) in the pancreatic tissue of hibernating 13-lined ground squirrels during torpor. This upregulation suggests a role for these miRNAs in the control of glucose metabolism and maintaining cellular homeostasis under low metabolic conditions characteristic of hibernation. The miR-29 family, including miR-29a-3p, has been shown to influence glucose metabolism and insulin signaling [31]. Studies have shown that elevated levels of miR-29a can negatively impact glucose uptake by reducing the expression of glucose transporter type 4 (GLUT4) in adipocytes [32]. However, in pancreatic β-cells, miR-29a is among the most abundant miRNAs and plays a crucial role in insulin secretion [33]. Knockout studies in mice have demonstrated that the absence of miR-29a leads to higher fasting blood glucose levels and reduced serum insulin, indicating its importance in maintaining normal glucose homeostasis [34]. The upregulation of miR-29a-3p during torpor may serve as an adaptive mechanism to regulate insulin secretion in response to the reduced metabolic demands of hibernation. By aiding in the regulation of insulin levels, miR-29a-3p could help maintain blood glucose within optimal ranges. This could functionally ensure sufficient energy supply to vital organs while conserving energy reserves in non-vital to survival tissue. The upregulations of miR-125a-5p and miR-22-3p have similarly been implicated in various relevant cellular processes. miR-125a-5p has been identified in the past to play roles in tumor suppression, reduction in oxidative stress, and apoptosis [35,36], while miR-22-3p has been similarly shown in several studies to be a tumor suppressor preventing cellular interactions [37,38,39]. The upregulation of these miRNA during torpor suggests a protective role in pancreatic tissues, which are likely subject to stress due to the repeated cycles of metabolic suppression and reactivation inherent in hibernation cycles. While specific studies on these miRNAs relevant to glucose metabolism are limited, their known functions in cellular stress responses indicate that their upregulation could help preserve pancreatic cell integrity during the torpor-arousal cycles. This preservation may be crucial for the resumption of normal pancreatic function upon arousal from torpor.

The observed downregulation of miR-21-5p and miR-148a-3p is consistent with a strategic suppression of insulin production and secretion during torpor, which reflects a broader reduction in metabolic activity. While studies have linked elevated levels of these miRNAs in circulation to diabetic phenotypes, these associations are primarily based on observational data [40]. The exact roles of these miRNA in metabolic regulation are thus not yet well characterized. Insulin is critical for regulating glucose uptake and storage, and its supposed downregulation based on pancreas inactivity during hibernation likely represents an adaptive mechanism to minimize unnecessary glucose utilization and energy expenditure [16]. This conservation strategy ensures that glucose remains available for critical functions, such as brain activity and cellular maintenance. miR-21-5p, which has an activity that is widely implicated in metabolic, proliferative, and inflammatory pathways, is markedly downregulated during torpor [41,42]. This reduction suggests a suppression of cellular growth and division pathways, aligning with the overall metabolic depression observed in hibernators. Moreover, given its involvement in inflammatory responses, the decrease in miR-21-5p may contribute to the anti-inflammatory state characteristic of hibernation, potentially protecting pancreatic tissue from damage during repeated torpor-arousal cycles [43]. However, it should be noted that the precise mechanisms by which miR-21-5p regulate these processes in the context of hibernation remain to be fully elucidated. Downregulated miR-148a-3p plays an important role in lipid metabolism and insulin secretion, aligning with the reduced demand for lipid mobilization and glucose uptake during metabolic rate depression [40,44]. Current studies suggest a link between miR-148a-3p and pancreatic beta-cell function [45]. A link between miR-200a-3p and the regulation of epithelial–mesenchymal transition and cell proliferation processes is known to exist. When suppressed, it is possible that this miRNA could contribute to a reduction in insulin release and cellular turnover [46,47]. miR-328-3p has also similarly been implicated in the regulation of cell proliferation and metabolic pathways [48]. The mechanistic details of these miRNA in the context of hibernation though are not well characterized, warranting further investigation.

The potential impact of these miRNA changes can be predicted using gene set analysis of both the GO terms and KEGG pathways affected. GO analysis indicated a marked suppression of terms associated with active cell signaling, adhesion, structural remodeling, and proliferation processes (Table A1). Analysis of GO molecular functions (MFs) revealed the downregulation of terms such as platelet-derived growth factor binding, protein and macromolecular complex binding, and extracellular matrix structural constituents. These terms are often viewed as integral to mediating cellular interactions (Figure 2). Similarly, GO biological process (BP) terms related to gene expression, including chromatin assembly, aging, vascular development, and cellular development processes, were repressed. Typically, these processes are engaged during periods of growth and repair (Figure 3). Downregulated GO cellular component (CC) terms included extracellular matrix components, cell–cell junctions, basement membrane, and somatodendritic compartments, terms involved in actively organizing and stabilizing tissue structure and facilitating intercellular communication (Figure 4).

Collectively, the coordinated downregulation of these GO terms (encompassing integrin binding, extracellular matrix remodeling, vascular development, and tissue repair) reflects a suppression of processes that are intimately associated with insulin signaling via shared mediators such as PI3K/Akt. For example, integrin engagement and focal adhesion dynamics are known to modulate insulin receptor activity and downstream signaling [49,50], while alterations in extracellular matrix composition can influence insulin sensitivity by affecting receptor accessibility [51]. Moreover, processes such as chromatin assembly and vascular development, which are critical for cellular growth and repair, have been shown to be energy-intensive and are suppressed under conditions that favor metabolic conservation, as observed during hibernation [52]. Together, these findings suggest that the downregulation of these GO terms contributes to an adaptive reduction in insulin signaling, thereby minimizing energy expenditure and prioritizing glucose allocation to vital organs under torpor conditions [53].

Additional KEGG pathway analysis depicts the absence of upregulated pathways in the pancreas of hibernating 13-lined ground squirrels, aligning with the metabolic suppression and cellular inactivity characteristic of torpor. During hibernation, energy conservation is a top priority [52,54]. This is achieved through a global downregulation of cellular processes, including transcription, translation, and signaling pathways that govern metabolism, proliferation, and stress responses [16,52,55]. The lack of upregulated pathways suggests that the pancreas operates at a minimal baseline level of activity, sufficient to maintain cellular integrity without investing energy into growth, repair, or endocrine functions such as insulin production. This global suppression likely reflects the reduced metabolic demands and the need to conserve energy during extended periods of low body temperature and reduced nutrient availability. Furthermore, the absence of pathway activation suggests a strong physiological shift toward dormancy, where the downregulation of energy-intensive processes outweighs any requirement for upregulation, supporting the survival strategy during hibernation.

The most significant downregulated KEGG pathways include the focal adhesion pathway, neurotrophin signaling pathway, and oncogenic pathways; pathways intrinsically linked to cellular energy metabolism (Figure 5). During hibernation, the pancreas of the 13-lined ground squirrel enters a state of suppressed activity as part of an energy-conserving strategy [1]. Consequently, cellular processes in the pancreas, such as proliferation, migration, and repair, are significantly reduced. This suppression directly impacts the focal adhesion pathway, which regulates cell adhesion, cytoskeletal remodeling, and signaling through integrins, focal adhesion kinase (FAK), and downstream PI3K/Akt and MAPK pathways [56]. These processes are energy-intensive and believed to be non-essential during torpor, as pancreatic cells shift into a quiescent state, reducing the need for structural remodeling and cell migration. This pathway is interconnected with the insulin signaling pathway through shared signaling molecules, particularly the PI3K/Akt pathway, which regulates cell survival, metabolism, and glucose uptake [57,58,59]. The insulin signaling pathway is critical for glucose uptake and storage. By reducing insulin-mediated glucose uptake in peripheral tissues, this adaptation prioritizes glucose availability for vital organs like the brain, which predominantly relies on glucose. Such a shift not only conserves energy but also minimizes cellular energy expenditure associated with glucose transport and phosphorylation. Similarly, the neurotrophin signaling pathway is downregulated due to reduced neuronal and neuroendocrine activity during hibernation. Neurotrophins, such as NGF and BDNF, and their receptors (e.g., TrkA and TrkB) play a role in neuronal survival and pancreatic regeneration [60]. However, during torpor, the sympathetic nervous system is largely suppressed [61], leading to diminished neurotrophic signaling. As neuronal stimulation declines, it is possible that the pancreas no longer requires neurotrophin signaling to maintain neural input or promote regeneration.

The downregulation of cancer pathways is also a consequence of cellular quiescence. Cancer-related pathways such as PI3K/Akt, Ras/MAPK, and Wnt signaling are typically active in proliferating cells to support growth, DNA repair, and replication [62,63]. However, in the hibernating state, pancreatic cells enter the G0 phase of the cell cycle, characterized by minimal DNA replication and reduced transcriptional activity [2,64]. The quiescent state ensures that energy is not spent on cell division or repair, and it reduces the activation of oncogenic pathways. Moreover, the stress-tolerant state during hibernation, with low metabolic stress and reduced reactive oxygen species, allows a minimal requirement of DNA damage response mechanisms that often activate cancer-related pathways. Together, this downregulation of the focal adhesion, neurotrophin signaling, and cancer pathways in the pancreas of hibernating 13-lined ground squirrels is a coordinated, adaptive response to conserve energy and prioritize survival. It is believed that these pathways are interconnected, as focal adhesion signaling activates neurotrophin and oncogenic pathways through shared intracellular mediators like PI3K/Akt and MAPK. By suppressing these energy-consuming processes, the hibernating pancreas achieves a state of reduced cellular activity, ensuring metabolic resources are preserved during prolonged periods of torpor.

While our findings provide a comprehensive overview of miRNA expression changes in the pancreatic tissue of the 13-lined ground squirrel during hibernation, the functional implications of these differentially expressed miRNAs remain hypothetical. Our pathway enrichment analysis suggests potential regulatory roles in metabolic adaptation, stress resistance, and cellular maintenance, but further experimental validation is needed to confirm these effects. Future studies employing loss- or gain-of-function assays, miRNA target validation, and protein-level analyses will be essential to determine the direct molecular impact of these miRNAs on the pathways identified. These findings still, however, highlight the functional role of energetically cheap to produce and fast acting miRNA to aid in achieving and maintaining a low metabolic rate, and potential therapeutic parallels. Notably, miR-29a-3p has been implicated in insulin resistance and glucose metabolism, with previous studies showing its dysregulation in diabetic models. Similarly, miR-22-3p has been identified as a regulator of glucose uptake and pancreatic beta-cell survival. The observed differential expression of these miRNAs during torpor suggests a strategic metabolic adaptation that may help maintain glucose homeostasis in extreme conditions. Understanding these miRNA-regulated pathways in hibernators could inform novel therapeutic strategies for metabolic diseases such as type 2 diabetes. Future studies could explore targeted miRNA alteration as a potential approach for improving insulin sensitivity and metabolic stability in diabetic patients [65,66]. Additionally, the ability of hibernators to suppress reactivate metabolic processes without incurring long-term damage offers a unique model for studying conditions such as diabetes, obesity, and metabolic syndrome. For instance, the suppression of insulin production during torpor mimics certain aspects of insulin resistance, while the protective roles of miRNAs like miR-125a-5p could inform therapeutic strategies for mitigating oxidative stress in chronic metabolic diseases.

## 5. Conclusions

This study reveals that hibernating 13-lined ground squirrels exhibit miRNA-mediated regulation of pancreatic function during torpor. Differential expression of key miRNAs, such as upregulation of miR-29a-3p, miR-22-3p, and miR-125a-5p, coupled with downregulation of miR-21-5p and miR-148a-3p aligns with a coordinated suppression of energy-intensive cellular processes. GO and KEGG analyses demonstrated significant miRNA-targeted downregulation of pathways involved in cell adhesion, neurotrophin signaling, and oncogenic activity. These changes suggest an adaptive reduction in insulin signaling and conservation of energy by prioritizing glucose availability for vital organs. Our findings provide novel insights into hibernation biology and highlight potential therapeutic targets for metabolic disorders and improved organ preservation strategies. These adaptations emphasize hibernators’ resistance to physiological and environmental stresses and suggest new avenues for translational research in metabolic disease and preservation.

## Data Availability

All sequencing data are available on Sequence Read Archive (SRA; BioProject ID: PRJNA1219602. Data is contained within the article or Supplementary Material.

## Supplementary Materials

The following supporting information can be downloaded at: https://www.mdpi.com/article/10.3390/biom15050616/s1, Table S1: differential expression, Table S2: GO BP, Table S3: GO CC, Table S4: GO MF, Table S5: KEGG

## Appendix A

**Table A1:** Significantly enriched GO term statistical values.

Category	GO Term	Coefficient	FDR-Adjusted *p*-Value
Molecular Functions	Platelet-derived growth factor binding	−0.5504746	3.142731917 × 10^−15^
	Protein complex binding	−0.0942946	4.3737568447164 × 10^−6^
	Macromolecular complex binding	−0.0795339	4.373756844716 × 10^−6^
Biological Processes	Blood vessel morphogenesis	−0.1572424	1.711962697235 × 10^−7^
	Vasculature development	−0.1410867	1.711962697235 × 10^−7^
	Positive regulation of cell morphogenesis involved in differentiation	−0.1999583	6.1046556730018 × 10^−6^
Cellular Components	Extracellular matrix component	−0.2068074	5.424849580565 × 10^−5^
	Cell–cell junction	−0.1291901	5.424849580565 × 10^−5^
	Extracellular matrix	−0.1210426	0.00011302

## References

[1] Carey H.V., Andrews M.T., Martin S.L. (2003). Mammalian Hibernation: Cellular and Molecular Responses to Depressed Metabolism and Low Temperature. Physiol. Rev..

[2] Morin P., Storey K.B. (2009). Mammalian Hibernation: Differential Gene Expression and Novel Application of Epigenetic Controls. Int. J. Dev. Biol..

[3] Sonsalla M.M., Love S.L., Hoh L.J., Summers L.N., Follett H.M., Bojang A., Duddleston K.N., Kurtz C.C. (2021). Development of Metabolic Inflammation during Pre-Hibernation Fattening in 13-Lined Ground Squirrels (Ictidomys Tridecemlineatus). J. Comp. Physiol. B.

[4] Wang L.C.H., Wolowyk M.W. (2011). Torpor in Mammals and Birds. Can. J. Zoöl..

[5] Tessier S.N., Wu C.W., Storey K.B. (2019). Molecular Control of Protein Synthesis, Glucose Metabolism, and Apoptosis in the Brain of Hibernating Thirteen-Lined Ground Squirrels. Biochem. Cell Biol..

[6] Rouble A.N., Hefler J., Mamady H., Storey K.B., Tessier S.N. (2013). Anti-Apoptotic Signaling as a Cytoprotective Mechanism in Mammalian Hibernation. PeerJ.

[7] Biggar K.K., Wu C.W., Tessier S.N., Zhang J., Pifferi F., Perret M., Storey K.B. (2015). Primate Torpor: Regulation of Stress-Activated Protein Kinases during Daily Torpor in the Gray Mouse Lemur, Microcebus Murinus. Genom. Proteom. Bioinform..

[8] Capraro A., O‘Meally D., Waters S.A., Patel H.R., Georges A., Waters P.D. (2020). MicroRNA Dynamics during Hibernation of the Australian Central Bearded Dragon (*Pogona vitticeps*). Sci. Rep..

[9] Davis-Dusenbery B.N., Hata A. (2010). Mechanisms of Control of MicroRNA Biogenesis. J. Biochem..

[10] O’Brien J., Hayder H., Zayed Y., Peng C. (2018). Overview of MicroRNA Biogenesis, Mechanisms of Actions, and Circulation. Front. Endocrinol..

[11] Tüfekci K.U., Meuwissen R.L.J., Genç Ş. (2014). The Role of MicroRNAs in Biological Processes. Methods Mol. Biol..

[12] Ingelson-Filpula W.A., Storey K.B. (2023). Hibernation-Induced MicroRNA Expression Promotes Signaling Pathways and Cell Cycle Dysregulation in Ictidomys Tridecemlineatus Cardiac Tissue. Metabolites.

[13] Logan S.M., Storey K.B. (2021). MicroRNA Expression Patterns in the Brown Fat of Hibernating 13-Lined Ground Squirrels. Genomics.

[14] Rehman S., Storey K.B. (2024). Small RNA and Freeze Survival: The Cryoprotective Functions of MicroRNA in the Frozen Muscle Tissue of the Grey Tree Frog. Metabolites.

[15] Röder P.V., Wu B., Liu Y., Han W. (2016). Pancreatic Regulation of Glucose Homeostasis. Exp. Mol. Med..

[16] Wu C.W., Biggar K.K., Storey K.B. (2013). Biochemical Adaptations of Mammalian Hibernation: Exploring Squirrels as a Perspective Model for Naturally Induced Reversible Insulin Resistance. Braz. J. Med. Biol. Res..

[17] Du H., Zhao Y., Li H., Wang D.W., Chen C. (2021). Roles of MicroRNAs in Glucose and Lipid Metabolism in the Heart. Front. Cardiovasc. Med..

[18] Li K., Zhao B., Wei D., Wang W., Cui Y., Qian L., Liu G. (2020). MiR-146a Improves Hepatic Lipid and Glucose Metabolism by Targeting MED1. Int. J. Mol. Med..

[19] Pheiffer C., Dias S., Rheeder P., Adam S. (2018). Decreased Expression of Circulating MiR-20a-5p in South African Women with Gestational Diabetes Mellitus. Mol. Diagn. Ther..

[20] Dinesen S., El-Faitarouni A., Dalgaard L.T. (2022). Circulating MicroRNAs Associated with Gestational Diabetes Mellitus: Useful Biomarkers?. J. Endocrinol..

[21] Frerichs K.U., Kennedy C., Sokoloff L., Hallenbeck J.M. (1994). Local Cerebral Blood Flow during Hibernation, a Model of Natural Tolerance to “Cerebral Ischemia”. J. Cereb. Blood Flow Metab..

[22] McMullen D.C., Hallenbeck J.M. (2010). Regulation of Akt during Torpor in the Hibernating Ground Squirrel, Ictidomys Tridecemlineatus. J. Comp. Physiol. B.

[23] Zhang J., Hadj-Moussa H., Storey K.B. (2016). Current Progress of High-Throughput MicroRNA Differential Expression Analysis and Random Forest Gene Selection for Model and Non-Model Systems: An R Implementation. J. Integr. Bioinform..

[24] Sai Lakshmi S., Agrawal S. (2008). PiRNABank: A Web Resource on Classified and Clustered Piwi-Interacting RNAs. Nucleic Acids Res..

[25] Kalvari I., Nawrocki E.P., Argasinska J., Quinones-Olvera N., Finn R.D., Bateman A., Petrov A.I. (2018). Non-Coding RNA Analysis Using the Rfam Database. Curr. Protoc. Bioinform..

[26] Kozomara A., Birgaoanu M., Griffiths-Jones S. (2019). MiRBase: From MicroRNA Sequences to Function. Nucleic Acids Res..

[27] Li H., Handsaker B., Wysoker A., Fennell T., Ruan J., Homer N., Marth G., Abecasis G., Durbin R. (2009). The Sequence Alignment/Map Format and SAMtools. Bioinformatics.

[28] Law C.W., Chen Y., Shi W., Smyth G.K. (2014). Voom: Precision Weights Unlock Linear Model Analysis Tools for RNA-Seq Read Counts. Genome Biol..

[29] Ritchie M.E., Phipson B., Wu D., Hu Y., Law C.W., Shi W., Smyth G.K. (2015). Limma Powers Differential Expression Analyses for RNA-Sequencing and Microarray Studies. Nucleic Acids Res..

[30] Ward J.H. (1963). Hierarchical Grouping to Optimize an Objective Function. J. Am. Stat. Assoc..

[31] Massart J., Sjögren R.J.O., Lundell L.S., Mudry J.M., Franck N., O’Gorman D.J., Egan B., Zierath J.R., Krook A. (2017). Altered MiR-29 Expression in Type 2 Diabetes Influences Glucose and Lipid Metabolism in Skeletal Muscle. Diabetes.

[32] Kaur P., Kotru S., Singh S., Behera B.S., Munshi A. (2020). Role of MiRNAs in the Pathogenesis of T2DM, Insulin Secretion, Insulin Resistance, and β Cell Dysfunction: The Story so Far. J. Physiol. Biochem..

[33] Dey S., Kwon J.J., Liu S., Hodge G.A., Taleb S., Zimmers T.A., Wan J., Kota J. (2019). MiR-29a Is Repressed by MYC in Pancreatic Cancer and Its Restoration Drives Tumor Suppressive Effects via Downregulation of LOXL2. Mol. Cancer Res..

[34] Dooley J., Garcia-Perez J.E., Sreenivasan J., Schlenner S.M., Vangoitsenhoven R., Papadopoulou A.S., Tian L., Schonefeldt S., Serneels L., Deroose C. (2016). The MicroRNA-29 Family Dictates the Balance Between Homeostatic and Pathological Glucose Handling in Diabetes and Obesity. Diabetes.

[35] Vo D.T., Karanam N.K., Ding L., Saha D., Yordy J.S., Giri U., Heymach J.V., Story M.D. (2019). MiR-125a-5p Functions as Tumor Suppressor MicroRNA And Is a Marker of Locoregional Recurrence And Poor Prognosis in Head And Neck Cancer. Neoplasia.

[36] Dakir E.-H., Mollinedo F., Dakir E.-H., Mollinedo F. (2019). Genome-Wide MiRNA Profiling and Pivotal Roles of MiRs 125a-5p and 17-92 Cluster in Human Neutrophil Maturation and Differentiation of Acute Myeloid Leukemia Cells. Oncotarget.

[37] Wang X., Yao Z., Fang L. (2021). MiR-22-3p/PGC1β Suppresses Breast Cancer Cell Tumorigenesis via PPARγ. PPAR Res..

[38] Hu Y., Setayesh T., Vaziri F., Wu X., Hwang S.T., Chen X., Yvonne Wan Y.J. (2023). MiR-22 Gene Therapy Treats HCC by Promoting Anti-Tumor Immunity and Enhancing Metabolism. Mol. Ther..

[39] You Y., Tan J.-X., Dai H.-S., Chen H.-W., Xu X.-J., Yang A.-G., Zhang Y.-J., Bai L.-H., Bie P., You Y. (2016). MiRNA-22 Inhibits Oncogene Galectin-1 in Hepatocellular Carcinoma. Oncotarget.

[40] Grieco G.E., Cataldo D., Ceccarelli E., Nigi L., Catalano G., Brusco N., Mancarella F., Ventriglia G., Fondelli C., Guarino E. (2018). Serum Levels of MiR-148a and MiR-21-5p Are Increased in Type 1 Diabetic Patients and Correlated with Markers of Bone Strength and Metabolism. Noncoding RNA.

[41] Zhang D., Ran J., Li J., Yu C., Cui Z., Amevor F.K., Wang Y., Jiang X., Qiu M., Du H. (2021). MiR-21-5p Regulates the Proliferation and Differentiation of Skeletal Muscle Satellite Cells by Targeting KLF3 in Chicken. Genes.

[42] Sarkar J., Gou D., Turaka P., Viktorova E., Ramchandran R., Raj J.U. (2010). MicroRNA-21 Plays a Role in Hypoxia-Mediated Pulmonary Artery Smooth Muscle Cell Proliferation and Migration. Am. J. Physiol. Lung Cell Mol. Physiol..

[43] Zeboudj L., Sideris-Lampretsas G., Silva R., Al-Mudaris S., Picco F., Fox S., Chambers D., Malcangio M. (2023). Silencing MiR-21-5p in Sensory Neurons Reverses Neuropathic Allodynia via Activation of TGF-β–Related Pathway in Macrophages. J. Clin. Investig..

[44] Goedeke L., Rotllan N., Canfrán-Duque A., Aranda J.F., Ramírez C.M., Araldi E., Lin C.S., Anderson N.N., Wagschal A., De Cabo R. (2015). Identification of MiR-148a as a Novel Regulator of Cholesterol Metabolism. Nat. Med..

[45] Sun G., Qi M., Kim A.S., Lizhar E.M., Sun O.W., Al-Abdullah I.H., Riggs A.D. (2023). Reassessing the Abundance of MiRNAs in the Human Pancreas and Rodent Cell Lines and Its Implication. Noncoding RNA.

[46] Bracken C.P., Gregory P.A., Kolesnikoff N., Bert A.G., Wang J., Shannon M.F., Goodall G.J. (2008). A Double-Negative Feedback Loop between ZEB1-SIP1 and the MicroRNA-200 Family Regulates Epithelial-Mesenchymal Transition. Cancer Res..

[47] Gregory P.A., Bert A.G., Paterson E.L., Barry S.C., Tsykin A., Farshid G., Vadas M.A., Khew-Goodall Y., Goodall G.J. (2008). The MiR-200 Family and MiR-205 Regulate Epithelial to Mesenchymal Transition by Targeting ZEB1 and SIP1. Nat. Cell Biol..

[48] Luo X., Yang S., Zhou C., Pan F., Li Q., Ma S. (2015). MicroRNA-328 Enhances Cellular Motility through Posttranscriptional Regulation of PTPRJ in Human Hepatocellular Carcinoma. Onco Targets Ther..

[49] Hynes R.O. (2002). Integrins: Bidirectional, Allosteric Signaling Machines. Cell.

[50] Legate K.R., Wickström S.A., Fässler R. (2009). Genetic and Cell Biological Analysis of Integrin Outside-in Signaling. Genes. Dev..

[51] Williams A.S., Kang L., Wasserman D.H. (2015). The Extracellular Matrix and Insulin Resistance. Trends Endocrinol. Metab..

[52] Storey K.B., Storey J.M. (2004). Metabolic Rate Depression in Animals: Transcriptional and Translational Controls. Biol. Rev. Camb. Philos. Soc..

[53] Saltiel A.R., Kahn C.R. (2001). Insulin Signalling and the Regulation of Glucose and Lipid Metabolism. Nature.

[54] Rehman S., Storey K.B. (2024). Dynamics of Epigenetic Regulation in Dryophytes Versicolor Skeletal Muscle: Lysine Methylation and Acetylation Involvement in Metabolic Rate Depression. J. Therm. Biol..

[55] Lant B., Storey K.B. (2010). An Overview of Stress Response and Hypometabolic Strategies in Caenorhabditis Elegans: Conserved and Contrasting Signals with the Mammalian System. Int. J. Biol. Sci..

[56] Zhao X., Guan J.L. (2010). Focal Adhesion Kinase and Its Signaling Pathways in Cell Migration and Angiogenesis. Adv. Drug Deliv. Rev..

[57] Meyts P.D. (2016). The Insulin Receptor and Its Signal Transduction Network. Endotext.

[58] Huang D., Cheung A.T., Thomas Parsons J., Bryer-Ash M. (2002). Focal Adhesion Kinase (FAK) Regulates Insulin-Stimulated Glycogen Synthesis in Hepatocytes. J. Biol. Chem..

[59] Rondas D., Tomas A., Soto-Ribeiro M., Wehrle-Haller B., Halban P.A. (2011). Novel Mechanistic Link between Focal Adhesion Remodeling and Glucose-Stimulated Insulin Secretion. J. Biol. Chem..

[60] Huang E.J., Reichardt L.F. (2001). Neurotrophins: Roles in Neuronal Development and Function. Annu. Rev. Neurosci..

[61] Yang Y., Yuan J., Field R.L., Ye D., Hu Z., Xu K., Xu L., Gong Y., Yue Y., Kravitz A.V. (2023). Induction of a Torpor-like Hypothermic and Hypometabolic State in Rodents by Ultrasound. Nat. Metab..

[62] Bahar M.E., Kim H.J., Kim D.R. (2023). Targeting the RAS/RAF/MAPK Pathway for Cancer Therapy: From Mechanism to Clinical Studies. Signal Transduct. Target. Ther..

[63] He Y., Sun M.M., Zhang G.G., Yang J., Chen K.S., Xu W.W., Li B. (2021). Targeting PI3K/Akt Signal Transduction for Cancer Therapy. Signal Transduct. Target. Ther..

[64] Dias I.B., Bouma H.R., Henning R.H. (2021). Unraveling the Big Sleep: Molecular Aspects of Stem Cell Dormancy and Hibernation. Front. Physiol..

[65] Dalgaard L.T., Sørensen A.E., Hardikar A.A., Joglekar M.V. (2022). The microRNA-29 family: Role in metabolism and metabolic disease. Am. J. Physiol. Cell Physiol..

[66] Senese R., Cioffi F., Petito G., de Lange P., Russo A., Goglia F., Lanni A., Potenza N. (2019). miR-22-3p is involved in gluconeogenic pathway modulated by 3,5-diiodo-L-thyronine (T2). Sci. Rep..

